# Functional Hyperspectral Imaging by High-Related Vegetation Indices to Track the Wide-Spectrum *Trichoderma* Biocontrol Activity Against Soil-Borne Diseases of Baby-Leaf Vegetables

**DOI:** 10.3389/fpls.2021.630059

**Published:** 2021-02-24

**Authors:** Gelsomina Manganiello, Nicola Nicastro, Michele Caputo, Massimo Zaccardelli, Teodoro Cardi, Catello Pane

**Affiliations:** Consiglio per la ricerca in agricoltura e l'analisi dell'economia agraria, Centro di ricerca Orticoltura e Florovivaismo, Pontecagnano Faiano, Italy

**Keywords:** BCAs, *Sclerotinia sclerotiorum*, *Sclerotium rolfsii*, *Rhizoctonia solani*, *Diplotaxis tenuifolia*, *Lactuca sativa*, fresh-cutting vegetables, plant reflectance

## Abstract

Research has been increasingly focusing on the selection of novel and effective biological control agents (BCAs) against soil-borne plant pathogens. The large-scale application of BCAs requires fast and robust screening methods for the evaluation of the efficacy of high numbers of candidates. In this context, the digital technologies can be applied not only for early disease detection but also for rapid performance analyses of BCAs. The present study investigates the ability of different *Trichoderma* spp. to contain the development of main baby-leaf vegetable pathogens and applies functional plant imaging to select the best performing antagonists against multiple pathosystems. Specifically, sixteen different *Trichoderma* spp. strains were characterized both *in vivo* and *in vitro* for their ability to contain *R. solani, S. sclerotiorum* and *S. rolfsii* development. All *Trichoderma* spp. showed, *in vitro* significant radial growth inhibition of the target phytopathogens. Furthermore, biocontrol trials were performed on wild rocket, green and red baby lettuces infected, respectively, with *R. solani, S. sclerotiorum* and *S. rolfsii*. The plant status was monitored by using hyperspectral imaging. Two strains, Tl35 and Ta56, belonging to *T. longibrachiatum* and *T. atroviride* species, significantly reduced disease incidence and severity (DI and DSI) in the three pathosystems. Vegetation indices, calculated on the hyperspectral data extracted from the images of plant-*Trichoderma*-pathogen interaction, proved to be suitable to refer about the plant health status. Four of them (OSAVI, SAVI, TSAVI and TVI) were found informative for all the pathosystems analyzed, resulting closely correlated to DSI according to significant changes in the spectral signatures among health, infected and bio-protected plants. Findings clearly indicate the possibility to promote sustainable disease management of crops by applying digital plant imaging as large-scale screening method of BCAs' effectiveness and precision biological control support.

## Introduction

Baby leaf vegetables constitute the major ingredient of ready-to-eat salads, very appreciated worldwide by consumers looking for healthy diets rich in fibers and low in calories, with organoleptic and nutraceutical traits particularly enhanced in pigmented varieties. Currently, in Italy, which is among the top European producers of these crops, it is estimated that more than 4,500 hectares are devoted, both in tunnels and, marginally, in open field, to grow baby salads for the high convenience food chain (Morra et al., 2017). A rather large group of different leafy vegetable species are included under this appellation, although by far, wild rocket [*Diplotaxis tenuifolia* (L.) DC.] and baby lettuce (*Lactuca sativa* L. var. *acephala*) are the most extensively cultivated. Because of the intensive exploitation of soils, continuous cropping, cultivars susceptibility to pathogens and reduced use of synthetic fungicides, those crops are dramatically prone to several diseases occurring in the humid and temperate microclimate of the sprinkler-irrigated tunnels/fields (Caruso et al., 2018; Gilardi et al., 2018a,b; Gullino et al., 2019). The soil-borne fungi *Rhizoctonia solani* Kuhn, *Sclerotinia sclerotiorum* (Lib.) de Bary and *Sclerotium rolfsii* Sacc., belonging to the *Phylum* Basidiomycota, are parenchymatic, polyphagous, necrotrophic pathogens of different salad crops, causing huge economic losses and symptoms ranging from the simple rotting of the attacked organs to the damping-off. Their non-chemical counteraction is particularly requested under sustainable management systems pursuing the zero residues goal, while it is mandatory according to the organic farming rules (Giménez et al., 2019). To this scope, the integrated disease management people are exploring alternative approaches to synthetic fungicides, including the implementation of effective microbes able to control phytopathogenic attacks, referred as biological control agents (BCAs).

Soil microbiota represents a precious reservoir of biocontrol microorganisms to impact plant health, growth and productivity in agricultural applications. Several fungal species belonging to the genus *Trichoderma* (Ascomycota) are known to suppress soil-borne and foliar plant diseases directly by mechanisms against the host pathogen (competition for space and nutrients, antibiosis, and mycoparasitism) and indirectly by the induction of a resistance responses in the colonized plants (Howell, 2003). Because of their crucial role as antagonists, *Trichoderma* spp. are among the most effective and commercialized biological control agents, registered as Plant Protection Products to manage a broad-spectrum of plant pathogens (Sharma et al., 2019). A number of *Trichoderma* spp. antagonistic strains are sourced from several telluric environments carrying disease control-related functions, including suppressive composts, to gain increasing efficacy firstly due to the niche-competence shared with the targeted soil-borne pathogens (Wang et al., 2019). The selection of novel and effective BCAs requires fast and robust screening methods suitable to evaluate high numbers of candidates. In this context, digital technologies, such as remote sensing, could play a pivotal role not only for early disease detection but also for the rapid performance analyses of BCAs and in the prediction of the biocontrol efficacy.

Hyperspectral imaging is a non-destructive and powerful digital technology to directly identifying biochemical and physiological shifts occurring in plants in response to external stimuli, including pathological prodding (Thomas et al., 2018). It involves the pixel-by-pixel analysis of an image containing spatially distributed the reflectance spectrum captured in the visible (VIS, spectral range 400–700 nm) and near infrared (NIR 700–1,000 nm) regions as hypercube dataset resulting by the interaction of the canopy with the incident light (Liu H. et al., 2020). Several previous hyperspectral studies pointed up broad/narrow extracted band indices, called vegetation indices (VIs) that have been used to associate the spectral information to several crop characteristics (Thenkabail et al., 2000), including plant health (Xue and Su, 2017). For example, the best known one, Normalized Difference Vegetation Index (NDVI) that is predictive of the vegetative growth and the general plant status (Rouse et al., 1973), recently was also proposed to refer about the *Vitis vinifera* – *Botrytis cinerea* interaction (Pañitrur-De la Fuente et al., 2020). The sensitivity of hyperspectral VIs about disease grade of the canopy, was also proposed to automatically evaluate the performances of disease control methods as innovative functional application (Martins et al., 2018). In this view, hyperspectral imaging may additionally help the fine scouting of new effective microbial antagonists under selection by configuring a standard quantitative analytic method to follow biocontrol dynamics that can be usefully implemented in a perspective definition of precision biological control guidelines.

The aim of this work was to select new useful antagonistic strains of *Trichoderma* able to protect wild rocket and baby lettuce from deleterious soil-borne pathogens. *R. solani* and S. *sclerotiorum* infections are very diffuse among these cultivations while *S. rolfsii* is going emerging importance on baby-leaf because of its attitude to grow at high temperature regime, as under greenhouse. Additionally, computing the reflectance data from the canopy of the bio-treated plants, this study can lead to the identification of high-performing vegetative indices (VIs) functional to the large-scale evaluation of the biocontrol effectiveness and, furthermore, to discriminate between healthy and infected plants.

## Materials and Methods

### Isolation of *Trichoderma* Strains

The sixteen *Trichoderma* strains characterized here, were isolated from a high suppressive rocket and fennel-derived compost (Pane et al., 2020; Scotti et al., 2020) and stored in the fungal collection of CREA-Centro di ricerca Orticoltura e Florovivaismo (Pontecagnano Faiano, Italy CREA-OF). Isolates were subjected to monosporic culturing by serial ten-fold dilution. For the strain characterization, macroscopic features (medium pigmentation, colony color, colony edge shape, smell) were evaluated after 7 days of growth on potato dextrose agar (PDA, Condalab, Madrid, Spain) medium at 25°C. Microscopic parameters (conidium length, width and shape) were also measured under light microscopy at 40× magnification with the optical microscope (Nikon Eclipse 80i, Nikon, Melville, NY, USA) in 0.05% Tween® 20 considering *n* = 40 conidia. All the isolates were maintained on PDA at 4°C and sub-cultured weekly.

### Identification of *Trichoderma* Strains

Isolates were grown in potato dextrose broth (PDB, Condalab, Madrid, Spain) on a rotary shaker at 120 rpm for 96 h at 25°C. Fresh mycelium was collected after vacuum filtration through No. 4 Whatman filter paper (Whatman Biosystems Ltd., Maidstone, UK), then frozen in liquid nitrogen, ground to a fine powder and immediately processed. Total genomic DNA was extracted from 100 mg of ground mycelium by using the PureLink® Plant Total DNA Purification Kit (Invitrogen™, ThermoFisher Scientific, Waltham, MA, USA) according to the manufacturer's protocol. PCR amplification of internal transcribed spacers and translation elongation factor 1α (TEF1) was performed in a Biorad C1000 Thermal Cycler (Bio-Rad, Hercules, CA) following PCR program: denaturation at 96°C for 2 min; 35 cycles of denaturation at 94°C for 30 s; annealing at 55°C for 30 s; extension at 68°C for 75 s; final extension at 68°C for 10 min. Primers ITS1 (5′-CTTGGTCATTTAGAGGAAGTAA-3′) and ITS4 (5′-TCCTCCGCTTATTGATATGC-3′) were used to amplify a fragment (~0.6 kb) of rDNA including ITS1 and ITS2 and the 5.8S rDNA gene (White et al., 1990; Gardes and Bruns, 1993) while the 5′ portions of translation elongation factor 1α (~0.8kb) coding region and introns were amplified with primers TEF1-F (5′- ATGGGTAAGGARGACAAGAC- 3′) and TEF1-R (5′-GGARGTACCAGTSATCATGTT-3′), which prime within conserved exons (O'Donnell et al., 1998). Amplicons were separated by gel electrophoresis in 1% w/v agarose supplemented with SYBR Safe DNA Gel Stain (Invitrogen, Paisley, UK). Amplicon sizes were determined against a 100 bp DNA ladder (Invitrogen™, ThermoFisher Scientific, Waltham, MA, USA). PCR products were purified by PureLink™ PCR Purification Kit (Invitrogen™, ThermoFisher Scientific, Waltham, MA, USA) following the manufacturer's instructions, quantified with a NanoDrop™ system (NanoDrop Technologies Inc., Wilmington, DE, USA) and sent to Sanger sequencing.

### Phylogenetic Reconstruction

Phylogenetic relationships of the 16 *Trichoderma* strains were investigated based on ITS and TEF1 sequences. DNA sequences were blasted against the NCBI GenBank database using default parameters and then aligned with the more related *Trichoderma* isolates by the Clustal W algorithm (Thompson et al., 1994) with MEGA7 software (Kumar et al., 2016). Multiple alignments parameters were gap penalty = 10 and gap length penalty = 10. The default parameters (Ktuple = 2, gap penalty = 5, window = 4, and diagonals saved = 4) were used for the pairwise alignment. Final alignment adjustments were made manually in order to remove artificial gaps, as reported by Ospina-Giraldo et al. (1999). The analysis was conducted on the two gene partial sequences separately. Aligned sequences were then concatamerized to a total length of 1,667 nucleotides. The evolutionary history was inferred using the maximum likelihood method. The evolutionary distances were computed using the Tamura-Nei model. The confidence of the branching was estimated by bootstrap (BP) analysis with 1,000 replications (1000 BP). *T. atroviride* sequences DAOM 233966, DAOM 231423, DAOM 233456, *T. longibrachiatum* sequences DAOM 231854, DAOM 232019 and DAOM 231850 and *T. harzianum* sequences DAOM 233458, DAOM 232032 and DAOM 232055, were used as references. All sequences were deposited in GenBank under the accession numbers reported in Table 1.

**Table 1:** List of Trichoderma strains identified in this study with the GenBank accession numbers of the internal transcribed spacer (ITS) and translation elongation factor 1α (TEF1) sequences.

**Strains**	**ITS**	**TEF1**	**Species**
Ta100	MW191738	MW201694	*Trichoderma atroviride*
Ta104	MW191739	MW201695	*Trichoderma atroviride*
Ta104C	MW191740	MW201689	*Trichoderma atroviride*
Ta104S	MW191741	MW201690	*Trichoderma atroviride*
Ta105	MW191742	MW201692	*Trichoderma atroviride*
Ta116	MW191743	MW201693	*Trichoderma atroviride*
Ta117	MW191744	MW201691	*Trichoderma atroviride*
Tl35	MW191751	MW201701	*Trichoderma longibrachiatum*
Ta56	MW191737	MW201688	*Trichoderma atroviride*
TaIC12	MW191745	MW246311	*Trichoderma atroviride*
Tat11	MW191747	MW201697	*Trichoderma atroviride*
Tat3C1	MW191746	MW201696	*Trichoderma atroviride*
ThCB	MW191749	MW201699	*Trichoderma harzianum*
ThRP	MW191750	MW201700	*Trichoderma harzianum*
Th23	MW191748	MW201698	*Trichoderma harzianum*
Tl41	MW191752	MW201702	*Trichoderma longibrachiatum*

### *In vitro* Dual Confrontation Assay

The ability of the sixteen *Trichoderma* strains to contain the development of *R. solani, S. sclerotiorum* and *S. rolfsii in vitro*, was evaluated by the dual culture technique. These phytopathogenic fungi were stored in the fungal collection of CREA-OF, maintained on PDA slants. Mycelial plugs of 5-mm diameter, obtained from the periphery of 7-days old cultures of both pathogen and *Trichoderma* strains were placed simultaneously on the border of the plate (9 cm diameter), about 0.25 mm from the edges at opposite sides. The Petri dishes containing PDA medium inoculated only with the pathogen were used as reference controls. All plates were incubated at 25°C and the radial growth was recorded 7-days post-inoculation. The growth inhibition percentage was calculated by using the formula:

$$\begin{array}{l} \text{Growth inhibition }\left(\%\right)=100-\frac{C-T}{C} \end{array}$$

where C = pathogen radial growth in the control and T = pathogen radial growth of the in the dual culture.

### *In vivo* Biocontrol Activity Assays

The biocontrol activity of *Trichoderma* strains was assessed *in vivo* against *R. solani* on wild rocket, *S. sclerotiorum* on green baby lettuce and *S. rolfsii* on red baby lettuce.

One L flasks containing 150 g of common millet seeds were saturated with a 0.1 × PDB (w/w) and autoclaved. Flasks were then inoculated with 15 plugs 5 mm diameter obtained from one-week-old plates of each pathogen maintained on PDA, and incubated for 21 days at 25°C. At the end of incubation, the inoculum was ground and added to sterilized peat soil at the final concentration of 1% (w/w) for *R. solani* and *S. rolfsii*, and 2% (w/w) for *S. sclerotiorum*, respectively, according to the pathogen virulence. In the uninfected pots, non-inoculated common millet prepared as described above, was added. *Trichoderma* spp. spore suspensions were obtained from one-week-old cultures maintained on PDA at 25°C. For each isolate, the conidia were harvested by washing the plates with sterilized water using a sterile brush. The suspension was filtered and collected in a 50 mL Falcon® tube (Falcon, USA). The spore suspension concentration was measured by a Burker chamber (Brand, Germany) and adjusted at 1 × 10^7^ spore mL^−1^. Seeds of wild rocket cv. Tricia (Enza Zaden, Italy), green baby lettuce cv 166 (Sementi Dom Dotto, Italy) and red baby lettuce cv. Pamela (Maraldi, Italy) were sown in vermiculite-filled 500 mL bowls, germinated in the dark at 25°C and then maintained in a growth chamber at 22°C with a 12-h photoperiod. The irrigation was manually performed daily and a basal NPK mix liquid fertilization was applied twice a week. After 15 days, plants were transplanted in plastic pots (7 cm diameter and 100 mL volume capacity) filled with sterile peat, infected as described above. Each treatment consisted of three pots (replicates) containing 5 plants each for baby lettuces, and 10 plants *per* pot for rocket. After that, *Trichoderma* suspension treatments were applied by soil drenching reaching a final concentration of 1 × 10^6^ spore mL^−1^. Untreated infected pots and healthy pots were used as reference controls.

Pot distribution was arranged randomly in the growth chamber at the same conditions described above. After 7-days incubation, each pot was assessed for hyperspectral images, disease incidence (DI%) and severity index (DSI). DI was calculated as the percentage of plants with disease symptoms on the total. Disease severity was assessed using a 1–3 scale adapted from Larkin and Honeycutt (2006): 0: no symptom; 1: foliar discoloration; 2: plant withering and visible lesion(s); 3: severe infection and plant dead.

DI% and DSI were calculated according to Yang et al. (2009). The experiment was performed twice.

### Hyperspectral Imaging

Hyperspectral images were acquired by using the SPECIM IQ camera (Specim, Spectral Imaging Ltd., Oulu, Finland) working in the range of 400–1,000 nm on a total of 204 wavelengths with a spectral resolution of 4 nm. The camera carries a CMOS sensor with a spatial sampling of 512 pixels and an image resolution of 512 × 512 pixel. The pixel size is 17.58 × 17.58 μm. Reflectance value was calculated automatically by the camera software. The images were captured under natural light conditions (Irradiance range 800–1,000 W/m^2^). One image *per* replicate (pot) was acquired, each containing all conditions (treatments) analyzed. Relative reflectance of hyperspectral images was simultaneously computed by the camera software. White reference, dark frame and raw data, were acquired during the measurements. The equation applied for the computation of the raw reflectance was as follows:

$$\begin{array}{l} \text{Raw Reflectance}=\frac{{\left(\text{Raw data}\right)}^{t1}-{\left(Dark\right)}^{t1}}{{\left(\text{White}\right)}^{t2}-\;{\left(Dark\right)}^{t2}}\;\times \;\frac{t2}{t1} \end{array}$$

where *White* is the white reference, *t1* and *t2* are integration times (used for a highly reflective white reference), and *Dark* represents a target with low reflectance.

**Figure 1:**
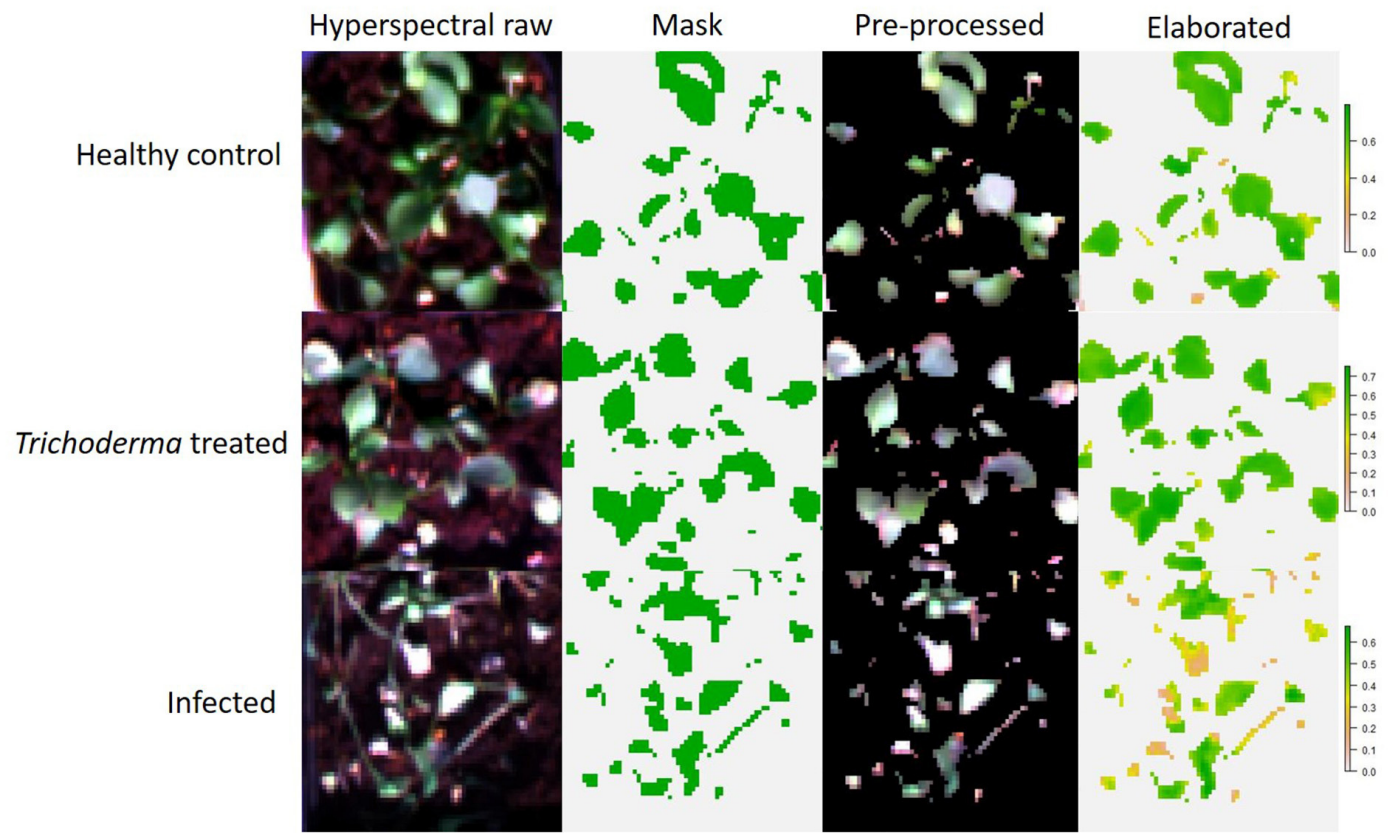
Workflow of data processing in hyperspectral imaging.

The elaboration of the hyperspectral images was carried out with the R software. Raster R package (Hijmans et al., 2015) was used to visualize and extract the hypercube dataset, successively elaborated into a typical spectral graphic. The unsupervisioned classification of the images was performed with Cluster R package to remove background once separated the objects “X” into “K” clusters. K-means clustering algorithm is a partitional or non-hierarchical clustering method (MacQueen, 1967; Anderberg, 1973), that here highlighted two clusters, background and plants (Figure 1). Then, the background cluster was deleted from the image, while the plant cluster was submitted to the extraction of the 46 hyperspectral VIs by imaging, averaging the pixel values for each replicate *per* treatment.

### Hyperspectral Vegetation Indices and Statistical Analysis

Measurements of the pathogen growth inhibitions *in vitro*, disease incidence and disease severity percentages, were subjected to the statistical analysis by GraphPad Prism Software. Ordinary one-way ANOVA was applied to test the effects of the *Trichoderma* strains on the assessed parameters. In all cases, the statistical analysis of variance was corrected for multiple comparisons by the Bonferroni hypothesis test, considering a *p*-value ≤ 0.05. Since experiment effect was not observed, data from the repeated experiments were pooled.

The same procedure was applied to evaluate the indices calculated on the hyperspectral dataset. Moreover, in order to select the most informative ones, they were analyzed, in relation to the observed disease severity in each pathosystem, by Multiple Variable analysis, applying the Pearson's correlation coefficient. The high-performing VIs that resulted commons to all the three host-pathogen target systems, were filtered on the base of a stringent statistical grid (*p*-value ≤ 0.05 and R^2^ > 0.5) and highlighted by using Venn diagram (http://bioinformatics.psb.ugent.be/webtools/Venn/). The heatmap visualization and the hierarchical clustering analysis of the selected indices were obtained applying ClustVis online software (https://biit.cs.ut.ee/clustvis). Unit variance scaling was applied to rows and columns and they were clustered using correlation distance and average linkage. Furthermore, the Principal Component Analysis (PCA) of vegetative indices / disease index for each pathosystem was performed with the *pca* function of the R Factoextra package (Kassambara and Mundt, 2017). Data were log-normalized and disease severity index was converted to “factor” by grouping in classes according to the following 0–4 scale: 0 = 0 ≤ DSI ≤ 0.2; 1 = 0.21 ≤ DSI ≤ 0.4; 2 = 0.41 ≤ DSI ≤ 0.6; 3 = 0.61 ≤ DSI ≤ 0.8, 4 = 0.81 ≤ DSI ≤ 1. Then, *lm* function (R package) was applied to fit linear models.

## Results

### Colony and Conidium Morphological Characteristics

**Figure 2:**
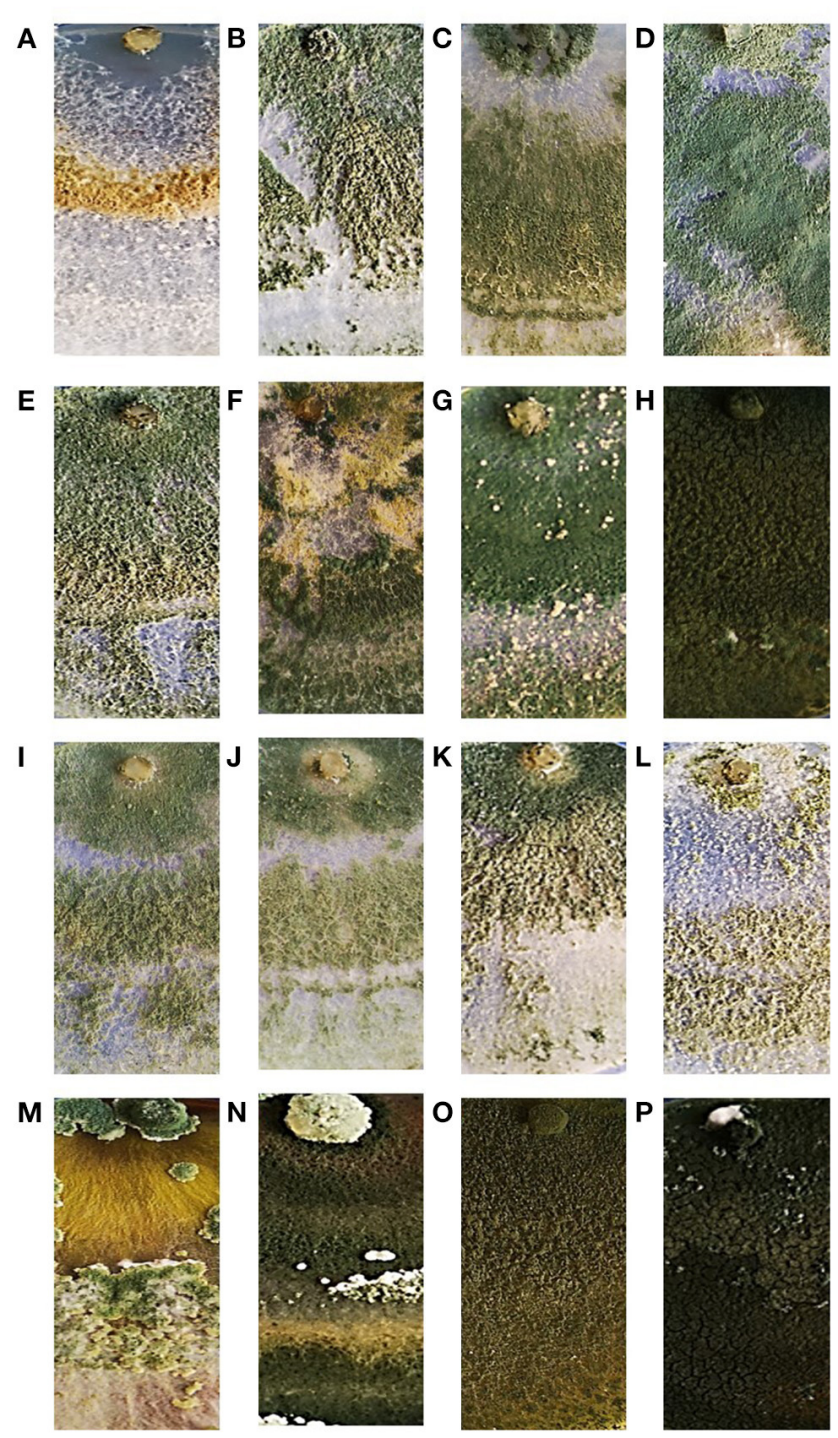
Colony appearance of the 16 *Trichoderma* strains used in this study after 7-days growth on PDA medium at 25°C. *T. atroviride* Ta100 **(A)**; *T. atroviride* Ta104 **(B)**; *T. atroviride* Ta104C **(C)**; *T. atroviride* Ta104S **(D)**; *T. atroviride* Ta105 **(E)**; *T. atroviride* Ta116 **(F)**; *T. atroviride* Ta117 **(G)**; *T. longibrachiatum* Tl35 **(H)**; *T. atroviride* Ta56 **(I)**; *T. atroviride* TaIC12 **(J)**; *T. atroviride* Tat11 **(K)**; *T. atroviride* Tat3C1 **(L)**; *T. harzianum* ThCB **(M)**; *T. harzianum* ThRP **(N)**; *T. harzianum* Th23 **(O)**; *T. longibrachiatum* Tl41 **(P)**.

The morphological characterization of the sixteen *Trichoderma* isolates studied in this work was carried out based on the inoculated medium appearance and pigmentation, color and edge of colonies, culture smell, shape and size of the conidia. After 5-days incubation at 25°C, the growth and sporulation patterns of the *Trichoderma* isolates showed significant differences. During the growth, due to the release of secondary metabolites, medium pigmentation varied significantly among the *Trichoderma* isolates, ranging from colorless to bright yellow and yellow-brownish to amber. Some of them showed a profuse production of conidia with coloration ranging from white to dark green (Figure 2). Furthermore, microscopic observations allowed highlighting differences in terms of conidia size and shape. In fact, the conidia of Ta56, Ta117, Ta105, ThRP, and Tat3C1 isolates, showed spherical shape with length-to-width ratio around 1, while the conidia of all the remaining strains, resulted ellipsoidal with length-to-width ratio > 1. The morphological colony and conidium features are summarized in Table 2.

**Table 2:** Main morphological features of colonies (medium pigmentation, color, edge and smell) and conidia (length, width, length-to-width ratio and shape) of the *Trichoderma* strains used in this study.

**Strains**	**Colonies**				**Conidia**			
	**Medium pigmentation**	**Color**	**Edge**	**Smell**	**Length (μm)**	**Width (μm)**	**L/W ratio**	**Shape**
Ta100	Uncolored	White	Smooth	-	4.5 ± 0.3	4.09 ± 0.2	1.13 ± 0.20	Ellipsoidal
Ta104	Uncolored	Dull green to bluish green	Smooth	-	3.9 ± 0.2	3.3 ± 0.3	1.22 ± 0.31	Ellipsoidal
Ta104C	Uncolored	Dull green to bluish green	Smooth	-	4.5 ± 0.3	4.1 ± 0.3	1.16 ± 0.22	Ellipsoidal
Ta104S	Uncolored	Dark green	Smooth	-	4.3 ± 0.3	3.77 ± 0.4	1.17 ± 0.16	Ellipsoidal
Ta105	Uncolored	Dull green to bluish green	Smooth	-	3.8 ± 0.3	3.8 ± 0.3	1.02 ± 0.21	Sphaeric
Ta116	Uncolored	Yellow to dark green	Smooth	-	4.5 ± 0.3	3.6 ± 0.4	1.27 ± 0.24	Ellipsoidal
Ta117	Uncolored	Dark green	Smooth	-	4.3 ± 0.2	4.2 ± 0.3	1.04 ± 0.21	Sphaeric
Tl 35	Yellow-greenish	Dark green-white tufts	Smooth	-	4.7 ± 0.5	3 ± 0.1	1.39 ± 0.28	Ellipsoidal
Ta56	Uncolored	Dull green to bluish green	Smooth	coconut	4,0 ± 0.4	4,1 ± 0,3	1.00 ± 1.15	Sphaeric
TaIC12	Uncolored	Dull green to bluish green	Smooth	coconut	4.6 ± 0.4	4.1 ± 0.3	1.17 ± 0.27	Ellipsoidal
Tat11	Uncolored	Dull green to bluish green	Smooth	coconut	5.4 ± 0.3	4.8 ± 0.2	1.14 ± 0.24	Ellipsoidal
Tat3C1	Uncolored	White-green	Wavy	-	4.2 ± 0.1	4 ± 0.2	1.09 ± 0.21	Sphaeric
ThCB	Amber	Scattered in tufts, yellow green	Wavy	-	3.7 ± 0.2	3.3 ± 0.2	1.14 ± 0.24	Ellipsoidal
ThRP	Yellow-brownish	Dark green	Smooth	-	4.9 ± 0.5	4.8 ± 0.5	1.05 ± 0.26	Sphaeric
Th 23	Bright yellow	Dark green-white tufts	Smooth	-	4.1 ± 0.2	3 ± 0.4	1.41 ± 0.25	Ellipsoidal
Tl41	Yellow-orange	Dark green	Smooth	-	4 ± 0.4	3.5 ± 0.3	1.20 ± 0.32	Ellipsoidal

### Determination of *Trichoderma* Species

**Figure 3:**
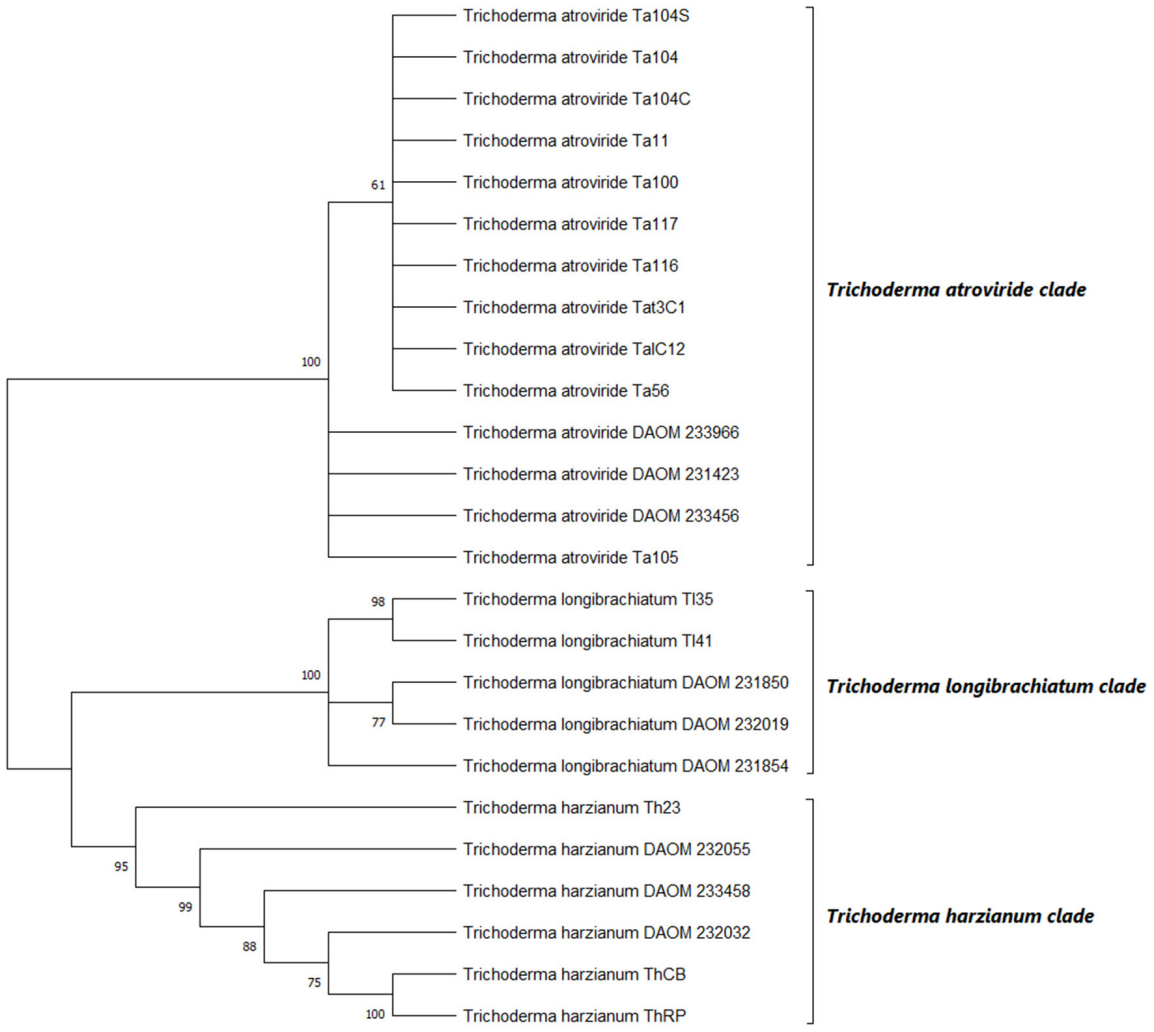
Phylogenetic relationships among the 16 strains of *Trichoderma* spp. inferred by analysis of rDNA (ITS) and translation elongation factor 1-α (TEF1) concatemers. The evolutionary history was inferred using the maximum likelihood method. The optimal tree is shown. The percentage of replicate trees in which the associated taxa clustered together in the bootstrap test (1000 replicates) are shown next to the branches. The evolutionary distances were computed by applying Tamura-Nei model. DAOM 233966. DAOM 231423 and DAOM 233456 (*T. atroviride*); DAOM 231854. DAOM 232019 and DAOM 231850 (*T. longibrachiatum*); DAOM 233458. DAOM 232032 and DAOM 232055 (*T. harzianum*) are the reference sequences.

The multi-locus sequence analysis is suggested for a better distribution of *Trichoderma* spp. in a phylogenetic tree (Samuels et al., 2010). Therefore, in the present work, concatemers of the ITS-TEF1 genes were used to contract the phylogenetic tree inferred by neighbor-joining method, as reported by Ospina-Giraldo et al. (1999). rDNA region and partial translated elongation factor locus amplifications, yielded products of ~600 and 800 bp, respectively, as estimated by agarose gel electrophoresis. Loci were analyzed separately, aligned and manually adjusted. Sequences were then grouped in concatamers and subjected to the phylogenetic analysis. This analysis involved 26 nucleotide sequences with a total of 1,667 positions in the final dataset. Based on the bootstrap values, the 16 *Trichoderma* strains were arranged into three distinct groups, belonging to *T. atroviride, T. longibrachiatum* and *T. harzianum* species (Figure 3). The strains Ta100, Ta104, Ta104C, Ta104S, Ta105, Ta116, Ta117, Ta56, Tat11, TaIC12, and Tat3C1, clustered within the *T. atroviride* clade, resulting aligned with the reference strains (DAOM 233966, DAOM 231423, and DAOM 233456). On the other hand, Tl35 and Tl41 strains were identified as *T. longibrachiatum*, showing a strictly association with the reference isolates (DAOM 231854, DAOM 232019, and DAOM 231850), while the strains Th23, ThRP and ThCB clustered in the *T. harzianum* clade. These identifications resulted well-supported by bootstrap tests, with values >60%.

### *In vitro* Dual Challenge Assay

**Figure 4:**
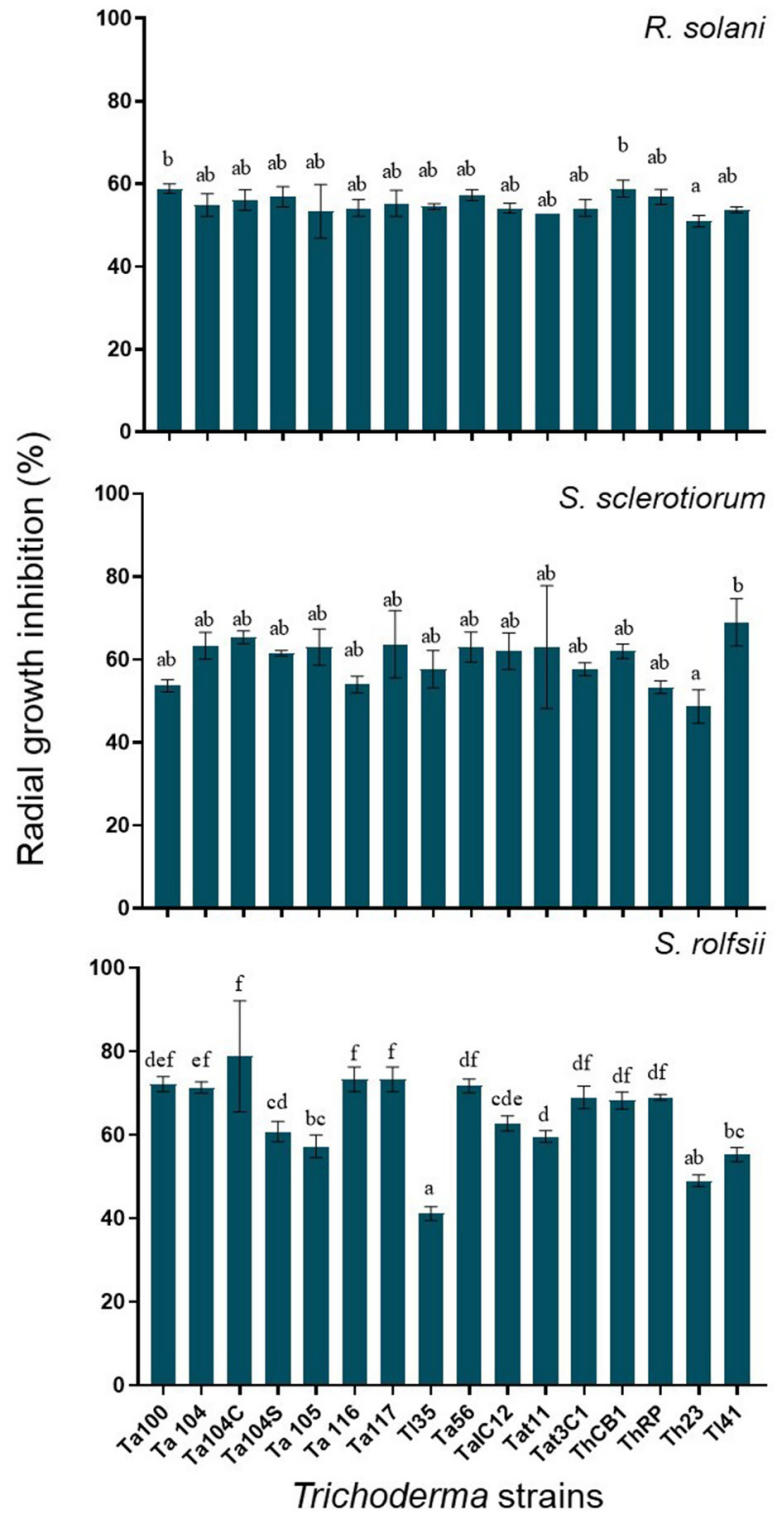
*R. solani*. *S. sclerotiorum* and *S. rolfsii* radial growth inhibition observed in dual culture assay. Values are expressed as inhibition percentage of pathogen radial growth. Each value is the average of three replicates. Bars with different letters are significantly different (*p*-value ≤ 0.05) according to ANOVA and Bonferroni correction test for multiple comparisons.

The dual culture assay was optimized to compare the inhibition activity of the 16 *Trichoderma* strains against the three soil-borne fungal pathogens. Since no significant differences were observed in the timing of growth among *Trichoderma* strains*, S. sclerotiorum, R. solani*, and *S. rolfsii*, the fungi were co-inoculated. As reported in Figure 4, all *Trichoderma* strains determined around 60% inhibition of *S. sclerotiorum* and *R. solani* radial growth. Only slight differences were observed among the different *Trichoderma* strains in inhibiting those phytopathogenic fungi. Furthermore, all the biocontrol strains, except Ta100 and Th23, reached the pathogen in 4–5 days and overgrew it in 9–10 days. On the other hand, most of the *Trichoderma* strains showed the ability to inhibit *S. rolfsii* radial growth up to 70%. Additionally, significant differences were observed among the different *Trichoderma* strains in containing this pathogen. In fact, a profuse overgrowth was observed for Ta116, ThRP, Ta105, Tat11, ThCB, Ta104C, Ta56, TaIC12, and Ta104S after 9 days, while Tl35 and Th23 resulted less effective in reducing the *in vitro* fungus development.

### *In vivo* Biocontrol Activity

**Figure 5:**
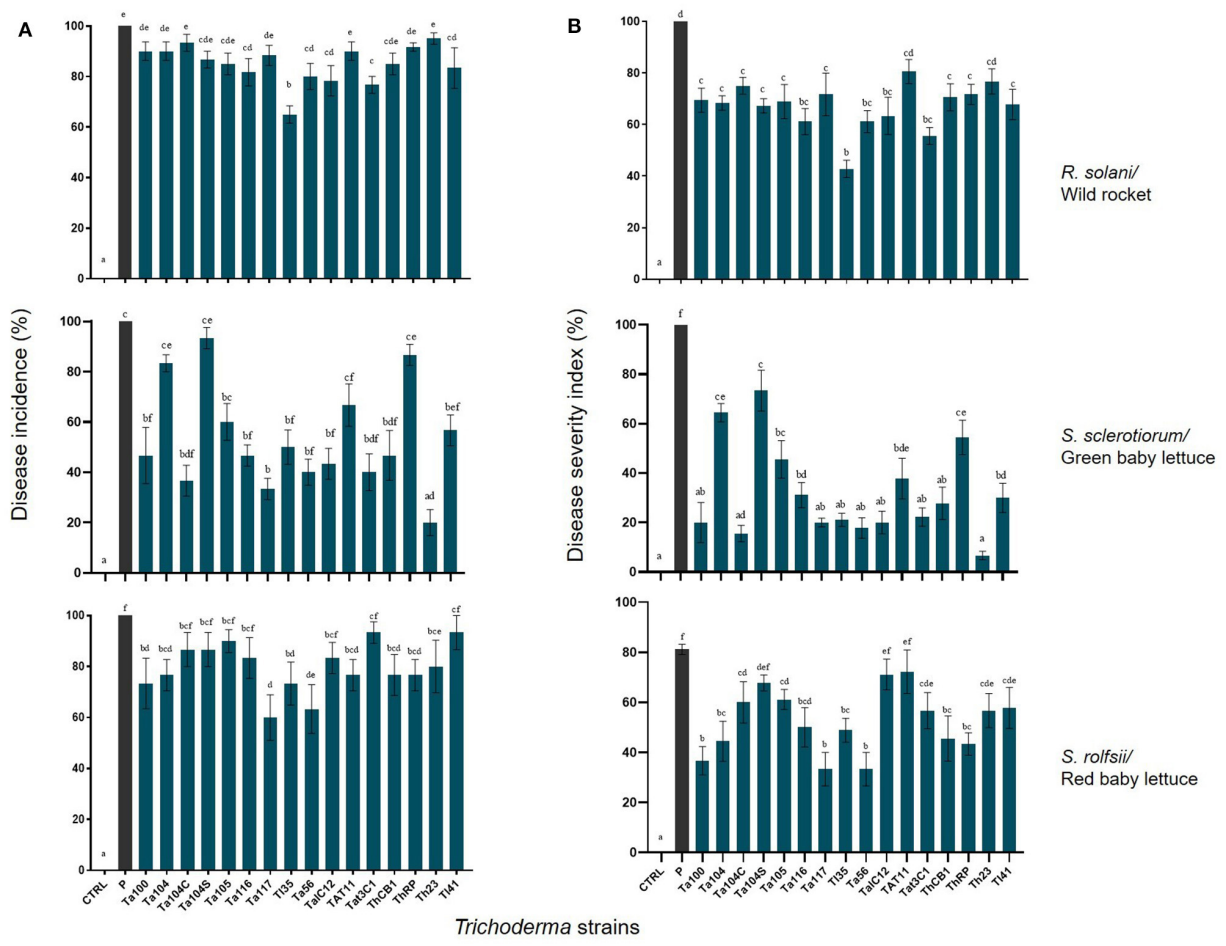
Disease incidence **(A)** and disease severity **(B)** percentages observed on *Trichoderma* treated wild rocket. green baby lettuce and red baby lettuce infected with *R. solani*. *S. sclerotiorum* and *S. rolfsii*. respectively. compared with the infected untreated control. Bars with different letters are significantly different (*p*-value ≤ 0.05) according to ANOVA and Bonferroni correction test for multiple comparisons.

The ability of the different *Trichoderma* strains to protect plants was investigated by *in vivo* assays with *R. solani* on wild rocket, *S. sclerotiorum* on green baby lettuce and *S. rolfsii* on red baby lettuce. On all cases, disease incidence percentages (Figure 5 left) and disease severity index (Figure 5 right) were assessed. Overall, a significant *Trichoderma* treatment effect was found (*p*-value <0.001), as well as the interaction between factor *Trichoderma* strain × plant/pathogen system (*p*-value < 0.001). The application of Ta116, Tl35, Ta56, TaIC12, Tat3C1, and Tl41, on wild rocket significantly reduced the percentage of Rhizoctonia disease incidence detected 120 h post-inoculation, in comparison with the infected control. In fact, only the 60% of Tl35 treated plants showed disease symptoms; for all the other treatments, the disease incidence was around 80%. Interestingly, all *Trichoderma* strains, except for Tat11 and Th23, contained the severity of the disease: the bio-treated plants displayed mild disease symptoms or were almost healthy.

On the other hand, the BCAs reduced Sclerotinia disease incidence on green baby lettuce, excepted for Ta104, Ta104S and ThRP; the number of plants with symptoms was significantly lower than that observed in the infected control and a consistent reduction in the disease severity index was also observed.

*Trichoderma harzianum* Th23 resulted the best one in containing *Sclerotinia* disease development. The strains Ta100, Ta104, Ta117, Tl35, Ta56, Tat11, ThCB, ThRP, and Th23, were able to control *S. rolfsii* on red baby lettuce determining a meaningful reduction of disease incidence. Furthermore, all *Trichoderma* treated plants, excepted for Ta104S, TaIC12, and Tat11 interactions, showed a significant lower disease severity index than the infected control.

### Hyperspectral Imaging

**Figure 6:**
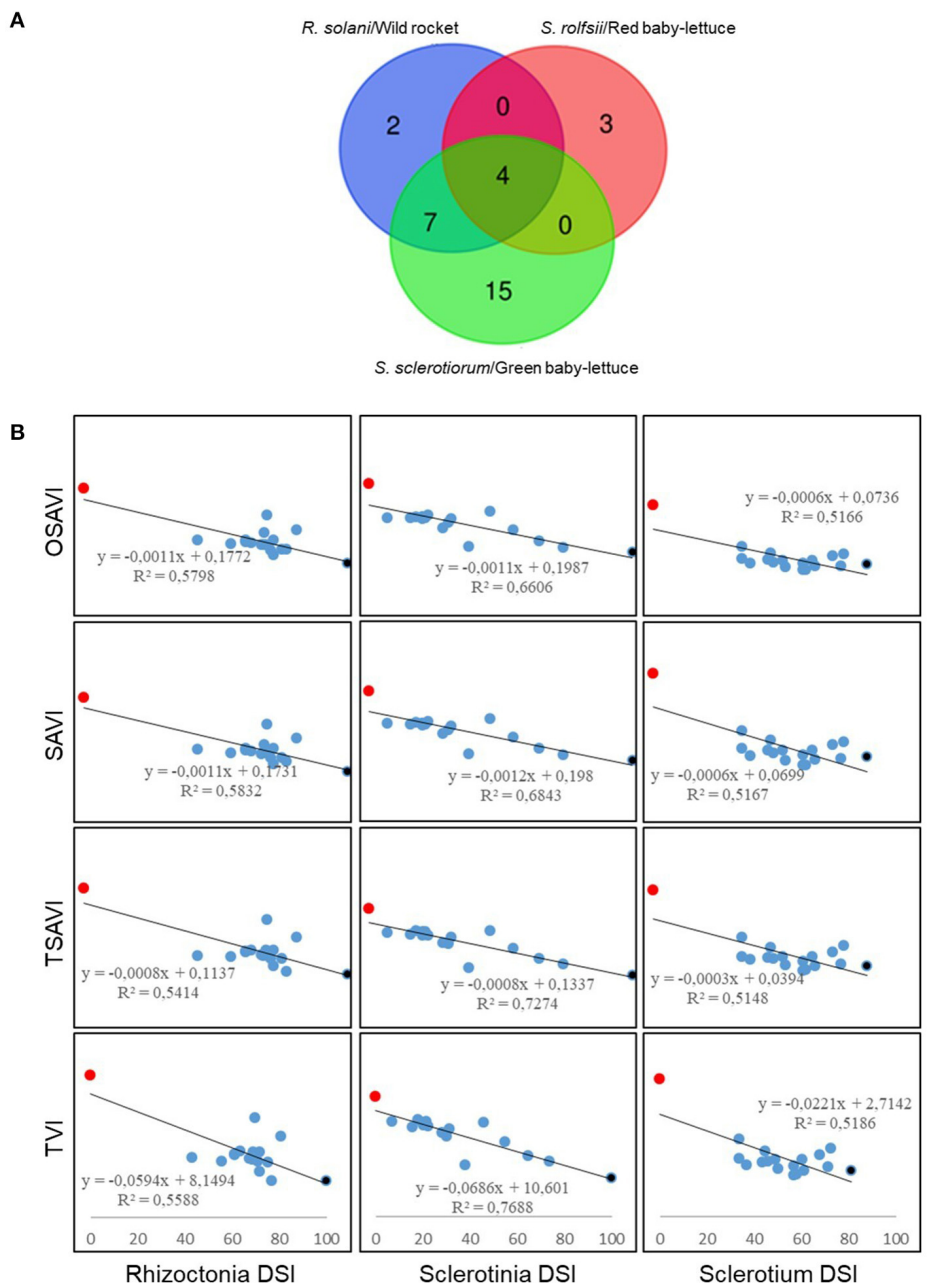
Selection of the significant (*p*-value ≤ 0.05 and R^2^ > 0.5) cross-correlated hyperspectral vegetation indices with disease severity index (DSI) for *R. solani*/wild rocket. *S. sclerotiorum*/green baby-lettuce and *S. rolfsii*/red baby-lettuce compatible interactions. **(A)** Venn diagram showing the number of the vegetative indices significantly cross-correlated with disease severity. common and not common to all the pathosystems. **(B)** Pearson's correlation between the common hyperspectral vegetative indices (OSAVI. SAVI. TSAVI and TVI) and DSIs. Non-inoculated healthy controls (red); infected plants (black); infected plants treated with *Trichoderma* strains (blue).

**Figure 7:**
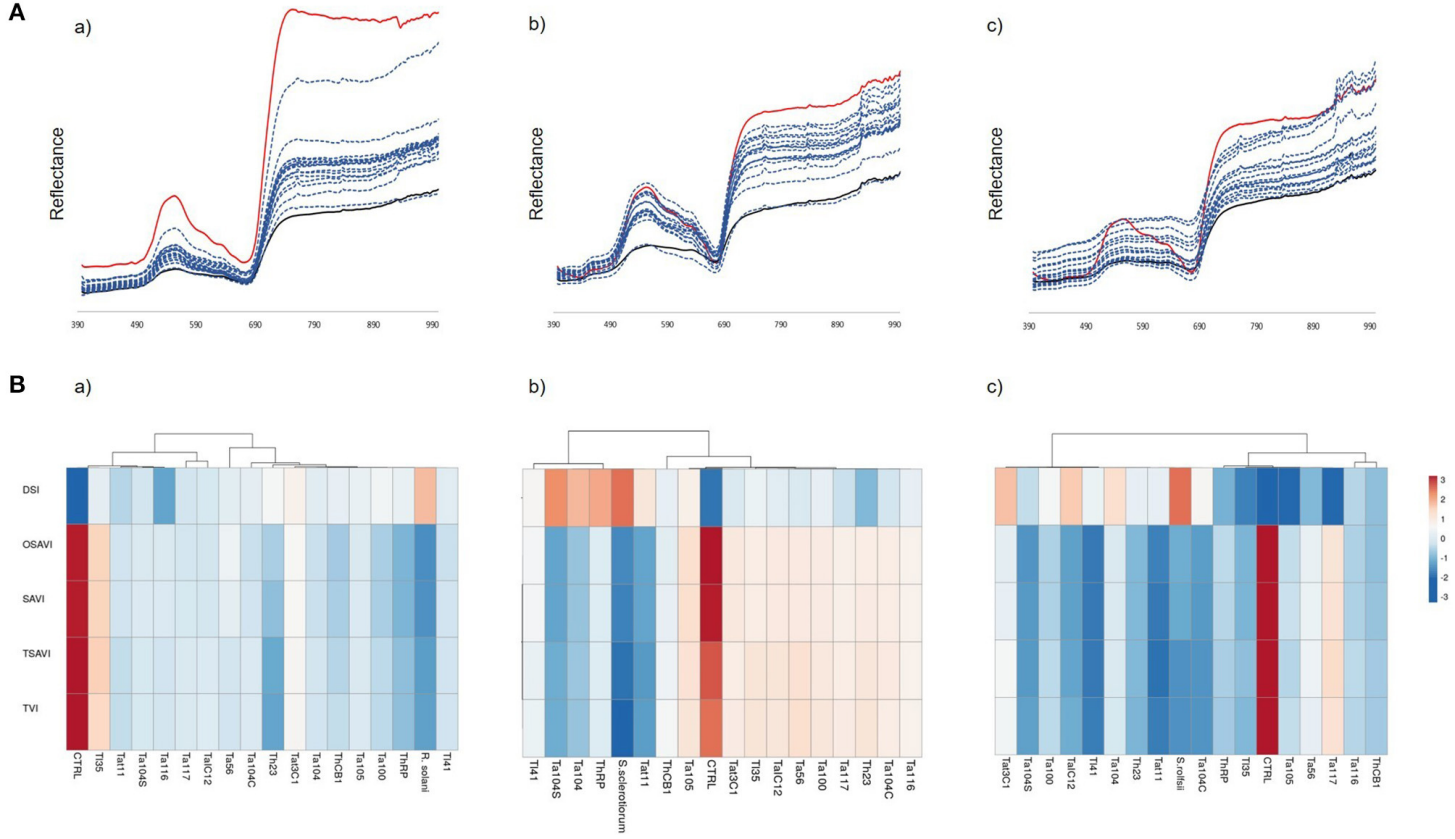
**(A)** Spectral signatures of wild rocket (a). green baby lettuce (b) and red baby lettuce (c) assayed with *R. solani*. *S. sclerotiorum* and *R. rolfsii*. respectively. and treated with the *Trichoderma* strains (blue). compared to the non-inoculated (red) and infected (black) controls. **(B)** Hierarchical clustering of OSAVI. SAVI. TSAVI. TVI in relation to the observed disease severity index (DSI) in the systems *R. solani*-wild rocket (a). *S. sclerotiorum*-green baby-lettuce (b). and *S. rolfsii*-red baby-lettuce (c). Rows were centered and unit variance scaling was applied. Columns were clustered using correlation distance and average linkage. Analysis was performed by ClustVis software.

Plants infected with the three soil-borne pathogens and exposed to the biocontrol treatment with *Trichoderma*, were subjected to hyperspectral imaging analysis in order to capture the spectral changes that occurred during the plant-pathogen-antagonist relation. As reported in Figure 5A, out of the 46 analyzed hyperspectral indices, 13 significantly cross-correlated with Rhizoctonia disease on rocket, 26 with Sclerotinia drop on green baby lettuce and 7 with Sclerotium rotting on red baby lettuce. Interestingly, four indices, OSAVI, SAVI, TSAVI and TVI, resulted shared by the three assayed pathosystems. The Multiple Variable analysis showed the score of their negative cross-correlation with the disease severity index for each plant/pathogen systems, with samples distributing between the two extremes, full healthy and full diseased (Figure 6B), coherently with changes visualized in the spectral signatures among non-inoculated, infected and infected but bio-treated plants (Figure 7A). Hence, heatmap visualization of the VIs/DSI hierarchical clustering quickly identified the most effective biocontrol agents in relation to the specific pathosystem (Figure 7B).

**Figure 8:**
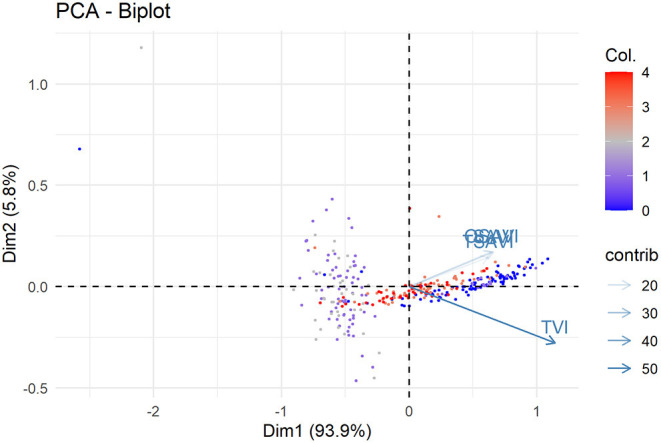
Principal component analysis of OSAVI. SAVI. TSAVI and TVI indices measured in wild rocket/*R. solani*. green baby lettuce/*S. sclerotiorum* and red baby lettuce/*S. rolfsii* pathosystems associated to the relative DSI. PC1 and PC2 explain the 93.9% and 5.8% of the total variability. respectively. Col indicate the distribution of DSI values in classes as follow: **0** = 0 ≤ DSI ≤ 0.2; **1** = 0.21 ≤ DSI ≤ 0.4; **2** = 0.41 ≤ DSI ≤ 0.6; **3** = 0.61 ≤ DSI ≤ 0.8. **4** = 0.81 ≤ DSI ≤ 1.

PCA analysis of VIs detected in the three different pathosystems showed their consistent ability to discriminate among different disease levels (Figure 8). Furthermore, OSAVI, SAVI and TSAVI resulted quite redundant, probably due to they differ only in the algorithm used for combining spectral data, while the distinct contribution in explaining the variance along PC1 (93.9%) was associated to TVI (Figure 8).

In order to fit a linear model, DSI data and selected indices were analyzed for multiple regression (Table 3). Based on the PCA results, SAVI indices (OSAVI, SAVI and TSAVI) computed together and TVI were submitted to linear regression analysis. OSAVI index was excluded since OSAVI:TVI interaction was found not significant in the resulting linear model. Results showed that F-statistic was highly significant (<3.5e-10) meaning that at least, one of the predictor is significantly related to the outcome variable. All the coefficients, including the interaction term coefficients, were statistically significant, suggesting that there is an interaction between the two predictor variables TSAVI + SAVI and TVI. On the other hand, these last are able to provide information about the biological observations although R-squared value was low. Thereby, statistical outputs corroborated the visualization by VIs images of the effects of *Trichoderma* strains on the disease symptom expressions over the cultivars.

**Table 3:** Summary of statistical values associated to linear regression model for prediction of DSI using SAVI (OSAVI, SAVI and TSAVI) and TVI vegetative indices.

	**Residuals**				
	**Min**	**1Q**	**Median**	**3Q**	**Max**
	−0.53359	−0.24166	−0.02817	0.22259	0.63078
	**Coefficients**				
	**Estimate**	**Std. Error**	**tvalue**	**Pr(>|t|)**	**Signif**.
(Intercept)	0.23261	0.05218	4.458	1.15E-05	^***^
TSAVI	−14.1965	4.65149	−3.052	0.002465	^**^
SAVI	10.91309	2.58815	4.217	3.24E-05	^***^
TVI	0.06265	0.02652	2.362	0.018777	^*^
TSAVI:TVI	1.56296	0.61288	2.55	0.011235	^*^
SAVI:TVI	−1.45497	0.4281	−3.399	0.000763	^***^
Residual standard error: 0.2812 on 318 degrees of freedom. Multiple R-squared: 0.1526. Adjusted R-squared: 0.1393. *F*-statistic: 11.46 on 5 and 318 DF. *p*-value: 3.5e-10.					

* P ≤ 0.05;

** P ≤ 0.01;

*** *P ≤ 0.001*.

**Figure 9:**
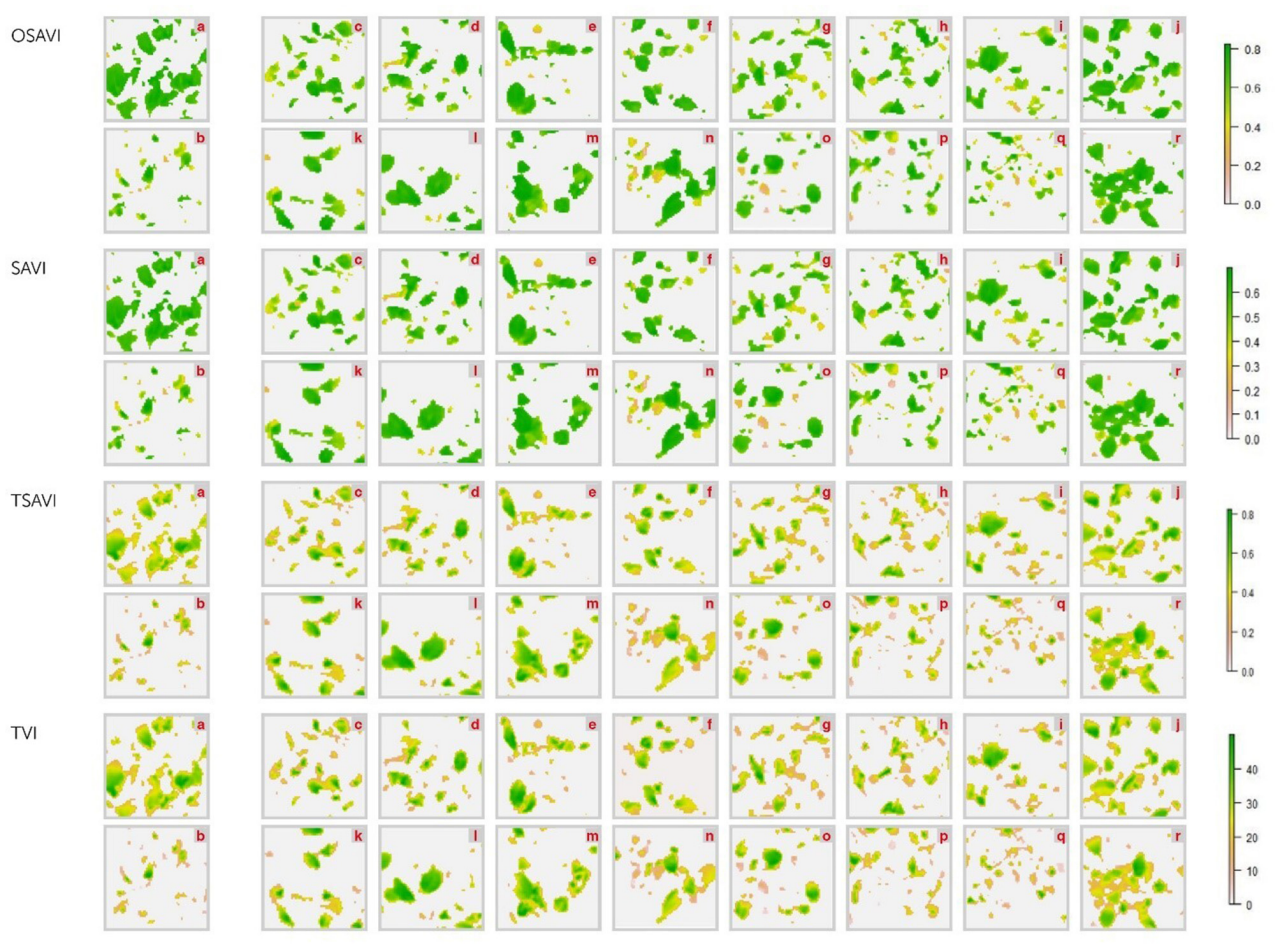
Hyperspectral vegetative indices (OSAVI. SAVI. TSAVI and TVI) images of wild rocket plants infected with *R. solani* and treated with *Trichoderma* strains (c–r) compared to non-infected (a) and infected (b) controls. acquired by Specim IQ hyperspectral camera. The description of the vegetative indices features are reported in Table 2. The list of *Trichoderma* treatments is as follow: *T. atroviride* Ta100 (c); *T. atroviride* Ta104 (d); *T. atroviride* Ta104C (e); *T. atroviride* Ta104S (f); *T. atroviride* Ta105 (g); *T. atroviride* 116 (h); *T. atroviride* Ta117 (i); *T. longibrachiatum* Tl35 (j); *T. atroviride* Ta56 (k); *T. atroviride* TaIC12 (l); *T. atroviride* Tat11 (m); *T. atroviride* Tat3C1 (n); *T. harzianum* ThCB (o); *T. harzianum* ThRP (p); *T. harzianum* Th23 (q); *T. longibrachiatum* Tl41 (r).

**Figure 10:**
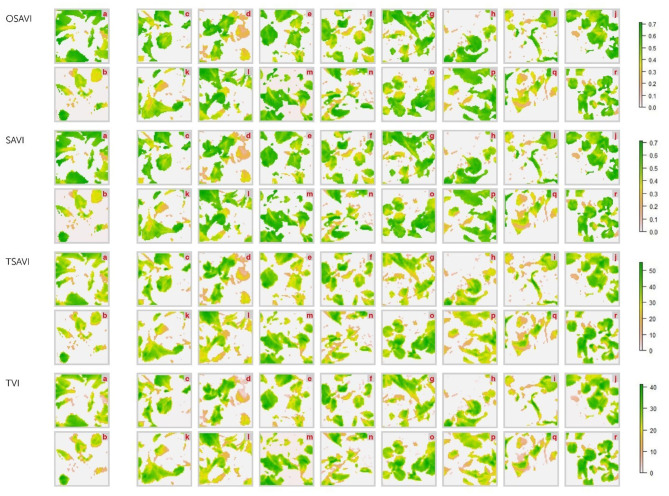
Hyperspectral vegetative indices (OSAVI. SAVI. TSAVI and TVI) images of green baby-lettuce plants infected with *S. sclerotiorum* and treated with *Trichoderma* strains (c–r) compared to non-infected (a) and infected (b) controls. acquired by Specim IQ hyperspectral camera. The description of the vegetative indices features are reported in Table 2. The list of *Trichoderma* treatments is as follow: *T. atroviride* Ta100 (c); *T. atroviride* Ta104 (d); *T. atroviride* Ta104C (e); *T. atroviride* Ta104S (f); *T. atroviride* Ta105 (g); *T. atroviride* 116 (h); *T. atroviride* Ta117 (i); *T. longibrachiatum* Tl35 (j); *T. atroviride* Ta56 (k); *T. atroviride* TaIC12 (l); *T. atroviride* Tat11 (m); *T. atroviride* Tat3C1 (n); *T. harzianum* ThCB (o); *T. harzianum* ThRP (p); *T. harzianum* Th23 (q); *T. longibrachiatum* Tl41 (r).

**Figure 11:**
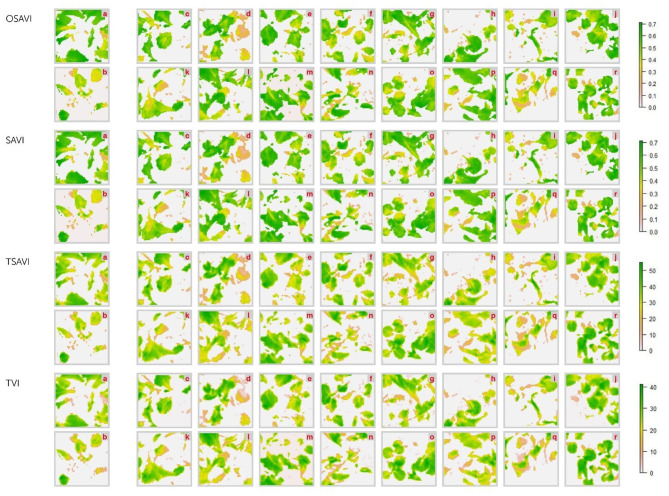
Hyperspectral vegetative indices (OSAVI. SAVI. TSAVI and TVI) images of red baby lettuce plants infected with *S. rolfsii* and treated with *Trichoderma* strains (c–r) compared to non-infected (a) and infected (b) controls. acquired by Specim IQ hyperspectral camera. The description of the vegetative indices features are reported in Table 2. The list of *Trichoderma* treatments is as follow: *T. atroviride* Ta100 (c); *T. atroviride* Ta104 (d); *T. atroviride* Ta104C (e); *T. atroviride* Ta104S (f); *T. atroviride* Ta105 (g); *T. atroviride* 116 (h); *T. atroviride* Ta117 (i); *T. longibrachiatum* Tl35 (j); *T. atroviride* Ta56 (k); *T. atroviride* TaIC12 (l); *T. atroviride* Tat11 (m); *T. atroviride* Tat3C1 (n); *T. harzianum* ThCB (o); *T. harzianum* ThRP (p); *T. harzianum* Th23 (q); *T. longibrachiatum* Tl41 (r).

Differences between healthy and diseased controls resulted, actually, perceptible on OSAVI, SAVI, TSAVI and TVI images, and the BCA treated plants displayed intermediate collocations (Figures 9–11). However, the correlational analysis identified disease-specific indices as reported in Figure 6A. Indeed, MCARI and SRPI resulted effective to track the *R. solani*/wild rocket interaction, other 15 indices (ARI, CAR, LRDSI, msr705, NDVI, PRI, PSSRc, R705, RDVI, RGRcn, RVI, RSVI, SIPI, TCARI, VARI-Green) were found significantly correlated to the *S. sclerotiorum* infection degree of green baby lettuce, while LIC3, VOG2, VOG3 were found suitable for following the *S. rolfsii*/red baby lettuce interaction. A summarization of Pearson's analysis involving all the VIs, is reported in Table 4.

**Table 4:** List of the hyperspectral vegetation indices from the literature that were used in this study for estimating their informative degree about disease severity.

**Index**		**Formula**	**Target**	**References**	**Significance on *R. solani*/Wild rocket**	**Significance on *S. sclerotiorum*/Green baby lettuce**	**Significance on *S. rolfsii*/Red baby lettuce**
AI	Anthocyanin index (AI)	(R_600_-R_699_)/(R_500_-R_599_)	Anthocyanin	Gamon and Surfus, 1999	ns	ns	ns
ARI	Anthocyanin Reflectance Index (ARI)	(1/R_550_)-(1/R_700_)	Carotenoids	Gitelson et al., 2001	ns	****	ns
CAR	Simple ratio 515/570	R_515_/R_570_	Carotenoids	Hernández-Clemente et al., 2012	ns	***	ns
DVI	Difference Vegetation Index	R_782_- R_675_	Plant vitality. Chlorophyll	Tucker, 1979	***	***	ns
FWBI1	Floating-position water band index (FWBI1)	R_900_/min (R_930_-R_980_)	Water	Harris et al., 2006	ns	ns	ns
FWBI2	Floating-position water band index (FWBI2)	R_920_/min (R_960_-R_1000_)	Water	Harris et al., 2006	ns	ns	ns
G	Simple Ratio 550/670 Greenness Index	R_550_/ R_670_	Plant vitality. chlorophyll	Smith et al., 1995	***	****	ns
GI	Greeness index	R_539_/ R_682_	Plant vitality	Zarco-Tejada et al., 2005	***	****	ns
Green- NDVI	Green Normalized Difference Vegetation Index	(R_750_- R_550_)/(R_750_+ R_550_)	Vegetation	Buschmann and Nagel, 1993	ns	ns	ns
HVI	Hyperspectral Vegetation Index	R_743_/ R_692_	Plant vitality	Gitelson et al., 1996	ns	ns	ns
LIC3	Simple Ratio 440/740 Lichtenthaler indices 3	R_440_/R_740_	Carotenoids	Lichtenthaler et al., 1996	ns	ns	***
LRDSI	Leaf rust disease severity index (LRDSI)	6.9 × (R_605_/R_455_)−1.2	Rust severity	Ashourloo et al., 2014b	ns	****	ns
MCARI	Modified Chlorophyll Absorption in Reflectance Index	R_712_x(R_712_-R_682_)−0.2x(R_712_- R_539_)] / R_682_	Chlorophyll	Daughtry, 2000	***	ns	ns
MCARI1	Modified Chlorophyll Absorption in Reflectance Index 1	1.2 x [2.5 x (R_800_- R_670_)−1.3 x (R_800_- R_550_)]	Plant vitality. Chlorophyll	Haboudane, 2004	ns	ns	ns
MSAVI	Modified Soil Adjusted Vegetation Index hyper	0.5x[2 x R_800_+ 1 –√(2 x R_800_+1)x 2 – 8 x (R_800_- R_670_)]	Healthy vegetation. Reduces soil noise	Qi et al., 1994	***	***	ns
mSR705	Modified Simple Ratio 705	(R_750_- R_445_)/(R_705_+ R_445_)	Vegetation	Wu et al., 2008	ns	****	ns
NDVI	Normalized Difference Vegetation Index (NDVI)	(R_800_-R_670_)/(R_800_+R_670_)	Plant vitality. Chlorophyll	Rouse et al., 1973	ns	****	ns
NPQI	Normalized Difference 415/435 Normalized Phaeophytinization Index	R_415_-R_435_/ R_415_+R_435_	Vegetation. Chlorophyll. Stress	Barnes et al., 1992	ns	ns	ns
OSAVI	Optimized Soil Adjusted Vegetation Index	(1+0.16) x(R_800_-R_670_) / (R_800_+ R_670_+ 0.16)	Relatively sparse vegetation	Rondeaux et al., 1996	***	****	***
PRI	Photochemical reflectance index (PRI)	(R_531_-R_570_)/(R_531_+R_570_)	Photochemical activity	Gamon et al., 1992	ns	****	ns
PRI515	Normalized Difference 515/531 Photochemical Reflectance Index 515/531	(R_515_- R_531_)/ (R_515_+R_531_)	Carotenoids	Hernández-Clemente et al., 2011	***	****	ns
PSSRa	Pigment-specific simple ratio (PSSR)	R_800_/R_680_	Chlorophyll a	Blackburn, 1998	***	***	ns
PSSRb	Pigment-specific simple ratio (PSSR)	R_800_/R_635_	Chlorophyll b	Blackburn, 1998	ns	ns	ns
PSSRc	Pigment-specific simple ratio (PSSR)	R_800_/R_470_	Carotenoids	Blackburn, 1998	ns	***	ns
PVI	Perpendicular Vegetation Index	(R_800_-0.2R_670_-0.6)/1.019	Vegetation	Wiegand and Hatfield, 1988	ns	ns	ns
R705/(R717+R491)	New vegetation index	R_705_/(R_717_+R_491_)	Vegetation. Chlorophyll	Tian et al., 2011	ns	***	ns
RARS	Simple Ratio 760/500 Ratio Analysis of Reflectance Spectra	R_746_/R_513_	Carotenoids	Chappelle et al., 1992	ns	ns	ns
RDVI	Renormalized Difference Vegetation Index	(R_800_-R_670_)/(R_800_+R_670_)^∧^0.5	Plant vitality. Chlorophyll	Roujean and Breon, 1995	ns	****	ns
Red Edge NDVI	Red Edge Normalized Difference Vegetation Index	R_750_-R_705_/ R_750_+R_705_	Vegetation	Gitelson and Merzlyak, 1994	ns	ns	ns
RGRcn	Red green ratio chlorophyll content (RGRcn)	(R_612_+R_660_)/(R_510_+R_560_)	Chlorophyll content	Steddom et al., 2003	ns	****	ns
RVI	Ratio vegetation index (RVI)	R_493_/R_678_	Plant vitality	Tilley et al., 2003	ns	****	ns
RVSI	Red-Edge Stress Vegetation Index	R_800_-R_670_/(R_800_+ R_670_)0.5	Plant vitality	Roujean and Breon, 1995	ns	****	ns
SAVI	Soil Adjustment Vegetation Index	((R_782_-R_675_)/(R_782_+R_675_+0.2)) (1.2)	Crop Parameters	Baret and Guyot, 1991	***	****	***
SIPI	Structure Intensive Pigment Index 1	R_800_- R_445_/R_800_/R_680_	Vegetation. Chlorophyll	Peñuelas et al., 1995	ns	***	ns
SRI	Simple Ratio Index	R_800_/R_680_	Vegetation. Chlorophyll. Leaf area index	Jordan, 1969	***	***	ns
SRPI	Simple Ratio Pigment Index (SRPI)	R_430_/R_680_	Vegetation. Chlorophyll	Peñuelas et al., 1994	***	ns	ns
TCARI	Transformed Chlorophyll Absorbtion Ratio	3 × [(R_700_-R_670_)−0.2 × (R_700_- R_550_) x R_700_/ R_670_)]	Plant vitality. Chlorophyll	Haboudane et al., 2002	ns	****	ns
TSAVI	Trasformed Soil Adjustment Vegetation Index	R_782_-R_675_	Crop Parameters	Baret and Guyot, 1991	***	****	***
TVI	Triangular Vegetation Index	0.5 × [120 x (R_750_-R_550_)−200 × (R_670_- R_550_)]	Plant vitality. Chlorophyll	Broge and Leblanc, 2001	***	****	***
VARIgreen	Visible atmospherically resistant index (VARIgreen)	(R_Green_-R_Red_)/(R_Green_+R_Red_-R_Blue_)	Vegetation	Gitelson et al., 2002	ns	****	ns
VOG1	Simple Ratio 740/720	R_740_/R_720_	Plant vitality. Chlorophyll	Vogelmann et al., 1993	ns	ns	ns
VOG2	Simple Ratio 734/747 Vogelmann indices	(R_734_-R_747_)/(R_715_+ R_726_)	Plant vitality. Chlorophyll	Vogelmann et al., 1993	ns	ns	***
VOG3	Simple Ratio 734/747 Vogelmann indices 3	(R_734_-R_747_)/(R_715_+ R_720_)	Plant vitality. Chlorophyll	Vogelmann et al., 1993	ns	ns	***
WBI	Water band index (WBI)	R_950_/R_900_	Water	Peñuelas et al., 1993	ns	ns	ns
WI	Water Index	R_900_/R_970_	Water	Peñuelas et al., 1997	ns	ns	ns
ZTM	Zarco - Tejada - Miller Index	R_750_/R_710_	Chlorophyll	Zarco-Tejada et al., 2001	ns	ns	ns

For each one the formula, functional target, literature reference and the significance level (ns: P > 0.05; *P ≤ 0.05; **P ≤ 0.01; ***P ≤ 0.001; ****P ≤ 0.0001) of the cross-correlation with disease severity index for the three different pathosystems were reported.

## Discussion

*Trichoderma* spp. include a plethora of isolates with biocontrol activity against phytopathogens (Kumar et al., 2017) that can also give additional benefits to the plants, such as increase the nutrient uptake, enhance the photosynthetic activity and stimulate different metabolic processes that positively affect yields and quality of the treated crops (El Enshasy et al., 2020). Recently, it has been shown that soil treatment with *Trichoderma* gave biostimulant effects on wild rocket and baby lettuce, ranging from the increase of leaf yield, fresh and dry weight, to the improvement of leaf nutritional status, resulting in a premium quality of the fresh-cuttings with higher lipophilic antioxidant activity and total ascorbic acid content (Fiorentino et al., 2018; Caruso et al., 2020; Di Mola et al., 2020; Rouphael et al., 2020). However, expressing the full biocontrol potential in these contexts, *Trichoderma*-based formulates can successfully integrate disease management protocols for producing baby leaf vegetables with high added value in terms of sustainability, decreasing the dependence on synthetic fungicides.

This study recruited sixteen new *Trichoderma* antagonistic strains assigned, on the base of the variations of the rRNA ITS and translation elongation factor 1-α gene partial sequences, to three different species, *T. longibrachiatum, T. atroviride*, and *T. harzianum*. Several stains of these species are well-known as BCAs of many pathogens affecting vegetables, including our targets (Bastakoti et al., 2017): they are proposed alone, being part of complex microbial consortia or activating suppressive organic amendments (Kareem et al., 2016; Wang et al., 2019; Chilosi et al., 2020). The macroscopic and microscopic examination of the selected strains showed interesting characters such as the profuse sporulation, the ability to secrete secondary metabolites in the medium changing its pigmentation and the capability of some to produce a volatile compound with the typical coconut-like aroma. This last specific character was detected in the strains Ta56, TaIC12, and Tat11 and could be putatively associated to the production of 6-pentyl-α-pyrone, a bioactive unsaturated δ-lactone with interesting properties involved in the microbial antagonism (Bonnarme et al., 1997; Serrano-Carreón et al., 2004; Longo and Sanromán, 2006; Ramos et al., 2008; Penha et al., 2012; Pascale et al., 2017). However, to clarify these aspects, further metabolomic investigations are necessary.

All the new identified antagonists significantly inhibited the mycelial growth of the pathogens in the dual culture assay. The main mechanism of control resulted to be the mycoparasitism, highlighted by the overgrowth of the BCAs onto the pathogen mycelia, observed already after 9–10 days of incubation. Mycoparasitism is one of the major weapons displayed by *Trichoderma* spp. against phytopathogens (Sachdev and Singh, 2020) allowing them to parasitize and kill the fungal host after the direct contact. During this intimate interaction, the beneficial fungus produces antibiotics and a huge array of cell degrading enzyme (protease, as β-glucanase, chitinase) necessary for the parasitism process (Steyaert et al., 2003).

*In vivo* biocontrol assays classified the *Trichoderma* BCA-candidates for the substantial ability to protect wild rocket, red and green baby lettuces from their most feared telluric fungal pathogens. Contrary to what was observed in *in vitro* assays, the *in planta* trials showed meaningful differences in biocontrol intensity among the strains in relation to the target pathosystem.

Specifically, Tl35, Ta56, Ta116, TaIC12, and Tat3C1 resulted the most effective strains in controlling Rhizoctonia damping-off of wild rocket, determining a significant reduction in terms of DSI (roughly 60%) compared with infected control under high disease pressure (100%). Rhizoctonia crown and root rot is a problematic disease of wild rocket for the ready-to-eat produces in the Italian cropping areas (Nicoletti et al., 2004). For their biological control, only the hyperparasite *Clonostachys rosea* has been noticed in literature (Nicoletti et al., 2007). Genetic resistance to this pathogen is not available yet (Pane et al., 2017), while wild rocket waste meals are proposed as amendments to promote the soil general suppressiveness providing antifungal molecules contained into the grounded plant tissues (Schlatter et al., 2017). Our results suggest that *Trichoderma* spp. can reduce the incidence and the severity of the disease and earn a chance as effective antagonist in Rhizoctonia damping-off management.

On the other hand, the biological control of *Sclerotinia* species, in particular *S. sclerotiorum*, has received increasing attention on adult lettuce inasmuch as chemical control of this pathogen is usually difficult, because the ascospores can infect any part of the head and sclerotia resist in the soil and can occur after prolonged wet periods (Patterson and Grogan, 1985; Elias et al., 2016; Subbarao et al., 2017). Therefore, the antagonists play a crucial role in the lettuce drop management because of their ability to parasitize the sclerotia in deep soil layers (Subbarao, 1998). As Sclerotinia drop biocontrol agents, *Coniothyrium minitans* Campbell, *Clonostachys rosea* (Schroers et al., 1999) and *Trichoderma* spp. resulted effective either in laboratory or in soil assays (Turner and Tribe, 1976; Phillips, 1989; Whipps and Budge, 1990; Jones et al., 2003, Bonini et al., 2020). Furthermore, against the compatible interaction between *Sclerotinia spp*. and adult lettuces, *Trichoderma* biocontrol agents have been reported to effectively reduce the seedlings drop (Elias et al., 2016), promote plant growth under infection (da Silva et al., 2019) and delay the symptoms appearance by emitting volatiles (da Silva et al., 2021). Accordingly, our results on baby lettuces demonstrated that *Trichoderma* strains Ta100, Ta104C, Ta117, Tl35, Ta56, TaIC12, Tat3C1, ThCB, and Th23, belonging to different species, can contain Sclerotinia drop disease, reaching a significant reduction of DSI (around 30%) compared to untreated control (DSI 100%).

Many previous studies reported the effectiveness of *Trichoderma* volatile compounds in inhibiting *S. rolfsii* growth (Hirpara et al., 2017; Marques et al., 2018; Sridharan et al., 2020). The culture filtrates with the highest antifungal effect against *S. rolfsii* were those of *T. brevicompactum* (Marques et al., 2018), *T. harzianum* (Saxena et al., 2014), *T. viride* (Darvin, 2013) and *T. virens* (Srinivasa and Devi, 2014). Wonglom et al. (2019) selected a *Trichoderma* strain with higher biocontrol properties against Sclerotium stem rot on red oak lettuce, due to its capability to produce volatile antifungal compounds (phenylethyl alcohol and epi-cubenol) and the cell wall degrading enzyme β-1,3-glucanase. Similarly, our results showed that *Trichoderma* strains Ta100, Ta104, Ta116, Ta117, Tl35, Ta56, ThCB1, and ThRP, highly suppressed lettuce Sclerotium rotting with a reduction of DSI around 50%, on average, compared to untreated infected control.

Interestingly, *T. atroviride* strain TA56 and *T. longibrachiatum* strain TA35 resulted to be multi-suppressive, namely highly effective in containing all the three diseases of the baby-leaf vegetables, demonstrating positive performances both *in vitro* and *in vivo*. The ability of these two BCAs to control the main disease of baby leaf make them promising candidates for a wide-spectrum application in preventive and/curative biological control practices in fresh-cut salad cropping, especially under soil sickness conditions.

Hyperspectral imaging was used here to objectively assess the biocontrol effectiveness of the comparing *Trichoderma* spp., interpreting the canopy reflectance response to the bio-treatments acquired by a hyperspectral sensor and summarized by VIs, thus, quantifying the functional effects. Four out of the 46 tried VIs, previously calibrated on plant physiological and structural shifts (Mishra et al., 2017), displayed significant (*p*-value < 0.05, highest coefficient of determination R^2^) positive relationships with plant health, as variably modulated by the biological control treatments contemporary in all the three target systems. The indices OSAVI, SAVI, TSAVI and TVI were able to highlight the most effective BCAs in controlling multiple soil-borne diseases of baby leaf vegetables. This result confirmed that selected indices can be applied as highly-informative tool for both BCA selections and disease monitoring in the presence of soil-borne pathogens generally associated to root and collar rot and, in advancing, leaf withering and plant death.

Therefore, the disease progression significantly affects the vegetation vitality and also the chlorophyll content. OSAVI, SAVI and TSAVI are soil adjusted vegetation indices, also defined as soil-line indices descriptive for sparse vegetation covering (Ren et al., 2018) as baby-leaf crops are. They have been used for grading wheat powdery mildew disease severity trough satellite-acquired scenes (Gröll et al., 2007; Feng et al., 2016; Ma et al., 2018). Recently, SAVI has been applied for the field estimation of the severity of cotton root rot caused by the fungus *Phymatotrichopsis omnivora* (Zhao et al., 2020), while OSAVI has been used to sense Fusarium Head Blight on wheat by computing Sentinel-2 multispectral data (Liu L. et al., 2020). Similarly to our findings, OSAVI has been found highly correlated with Rhizoctonia crown and root rot severity on sugar beet assessed with a non-imaging remote sensing approach (Reynolds et al., 2012). On the other hand, TVI is the triangular vegetation index associated to leaf chlorophyll content (Cui et al., 2019) and plant vitality (Broge and Leblanc, 2001). It has been calibrated for the leaf area index estimation (Xing et al., 2020) and is also known for describing spectral variations due to wheat leaf rust symptoms caused by *Puccinia triticina* (Ashourloo et al., 2014a,b).

To the best of our knowledge, this is the first study that retrieved hyperspectral VIs with high discriminatory capability for the biocontrol ability of *Trichoderma* against developing soil-borne diseases of leafy vegetables. Previously, Silva et al. (2018) have tried to apply a laser speckle based on a light signal at 632 nm to assess the efficacy of maize seed treatments with *T. harzianum* on the germination, vigor and sanitation of seedlings. Instead, Pishchik et al. (2016) have calculated VIs on VIS, RED (red-edge), NIR and MID (middle infrared) spectral information acquired with a field pulse photometer, to tentatively track the synergistic effect of the plant growth promoting bacteria, *Bacillus subtilis* and a humic fertilizer on lettuce plants quality and vitality.

The four indices of this study, each applying its own peculiar algorithm, work in the spectral range 550–800 nm, just on the border between VIS and NIR regions, suggesting that this part of the spectrum could be sensitive to the reflectance shifts occurring at canopy level during the plant-pathogen-antagonist interaction. Marín-Ortiz et al. (2020) have found in the VIS/NIR range 448–995 nm the distinctive spectral response of tomato to the *Fusarium oxysporum* infection that has been also associated to changes in the leaf concentration of chlorophyll and carotene. Similarly, the soil-borne pathogens studied in our systems could bring to the decline of chlorophyll and other pigments, as-well-as growth reduction conditioning the reflectance reaction.

As a matter of fact, decreases in chlorophyll content has been noticed in Rhizoctonia diseased carrot (Ahmad et al., 2019), in cucumber affected both by *R. solani* and *S. rolfsii* (Kotasthane et al., 2015) and in soybean attacked by *S. sclerotiorum* (Vitorino et al., 2020). On the contrary, *Trichoderma* can enhance the phothosynthetic performances of the colonized plants by increasing their chlorophyll content and, at the same time, determining an improvement of their general physiological status (Singh et al., 2013; Doley et al., 2014; Kotasthane et al., 2015) exerting an antagonistic action with respect to the pathogen in promoting the vitality of the plant. Therefore, according to these inferences the plant functional imaging as applied here may return valuable information about how the biocontrol agents is working.

Findings of the present study indicate the potential to boost the sustainability of disease management protocols trough high-performing hyperspectral VIs that can drive the biocontrol practices, such as, for example, the microbial augmentation, based on the early recognition of the worsening of the plant state and of the possible effectiveness reduction of the adopted plant protection strategy. Functional plant imaging can be used to track the plant progression under biocontrol effect using a restricted number of bands. The digital imaging has been proposed for the early diagnosis of plant diseases (Lowe et al., 2017), for the real-time field estimation of phytopathological conditions (Golhani et al., 2018) and to provide useful information for pest and disease control (Yao et al., 2011). Here, it helped to scout effective biological control agents against baby-leaf salad pathogens, demonstrating the potential to sense the biocontrol making on developing soil-borne diseases. The association between BCAs and hyperspectral imaging, concurring at reducing chemical pressure of fungicides on the environment and avoiding crop losses for uncontrolled pathogenic attacks, opens to the concept of precision biological control. The availability of digital tools for the automatized large-scale evaluation of biocontrol evolution will be useful both in field/greenhouse systems to rapidly assess the success of biological measures against phytopathogens as well as Susič et al. (2020) have recently pointed up for pest control.

## Conclusions

The high-effective *Trichoderma* strains identified in this study are able for protecting baby-leaf vegetables from a wide-spectrum of soil-borne pathogens, such as *R. solani, S. sclerotiorum*, and *R. rolfsii*. Strains belonging to *T. longibrachiatum, T. atroviride*, and *T. harzianum* are suitable for large-scale preventive applications in greenhouses that host wild rocket and baby-lettuces in succession and/or in rotation and have a perspective to work in consortia since they sourced from a unique niche. The scenario of applying digital imaging as innovative scheme to boost biological control, from the high throughput screening of the microorganisms to their field application, is highlighted. OSAVI, SAVI, TSAVI, and TVI, that were found highly correlated to disease severity, are promising and informative hyperspectral VIs to track biological control activity against multiple soil-borne pathogens of baby leaf vegetables. In future studies, digital imaging will be able to integrate metabolomic linked to transcriptomic analyses, which, supported by machine learning processing, can contribute to further improve the accuracy of the forecasting models by imaging applied to the plant protection practices.

## Data Availability Statement

The datasets presented in this study can be found in online repositories. The names of the repository/repositories and accession number(s) can be found below: https://www.ncbi.nlm.nih.gov/genbank/, MW191738; https://www.ncbi.nlm.nih.gov/genbank/, MW191739; https://www.ncbi.nlm.nih.gov/genbank/, MW191740; https://www.ncbi.nlm.nih.gov/genbank/, MW191741; https://www.ncbi.nlm.nih.gov/genbank/, MW191742; https://www.ncbi.nlm.nih.gov/genbank/, MW191743; https://www.ncbi.nlm.nih.gov/genbank/, MW191744; https://www.ncbi.nlm.nih.gov/genbank/, MW191751; https://www.ncbi.nlm.nih.gov/genbank/, MW191737; https://www.ncbi.nlm.nih.gov/genbank/, MW191745; https://www.ncbi.nlm.nih.gov/genbank/, MW191747; https://www.ncbi.nlm.nih.gov/genbank/, MW191746; https://www.ncbi.nlm.nih.gov/genbank/, MW191749; https://www.ncbi.nlm.nih.gov/genbank/, MW191750; https://www.ncbi.nlm.nih.gov/genbank/, MW191748; https://www.ncbi.nlm.nih.gov/genbank/, MW191752; https://www.ncbi.nlm.nih.gov/genbank/, MW201694; https://www.ncbi.nlm.nih.gov/genbank/, MW201695; https://www.ncbi.nlm.nih.gov/genbank/, MW201689; https://www.ncbi.nlm.nih.gov/genbank/, MW201690; https://www.ncbi.nlm.nih.gov/genbank/, MW201692; https://www.ncbi.nlm.nih.gov/genbank/, MW201693; https://www.ncbi.nlm.nih.gov/genbank/, MW201691; https://www.ncbi.nlm.nih.gov/genbank/, MW201701; https://www.ncbi.nlm.nih.gov/genbank/, MW201688; https://www.ncbi.nlm.nih.gov/genbank/, MW201697; https://www.ncbi.nlm.nih.gov/genbank/, MW201696; https://www.ncbi.nlm.nih.gov/genbank/, MW201699; https://www.ncbi.nlm.nih.gov/genbank/, MW201700; https://www.ncbi.nlm.nih.gov/genbank/, MW201698; https://www.ncbi.nlm.nih.gov/genbank/, MW201702; https://www.ncbi.nlm.nih.gov/genbank/, MW246311.

## Author Contributions

GM and CP conceived and designed the study and wrote the initial manuscript. GM, NN, MC, and CP conducted the experiments. GM and NN analyzed data. CP assisted in data analysis and interpretation of results. MZ and TC reviewed and edited the final version of the manuscript. All authors have read and agreed to the published version of the manuscript.

## Conflict of Interest

The authors declare that the research was conducted in the absence of any commercial or financial relationships that could be construed as a potential conflict of interest.
